# Multiparametric analysis of geometric features of fibrotic textures leading to cardiac arrhythmias

**DOI:** 10.1038/s41598-021-00606-x

**Published:** 2021-10-26

**Authors:** T. Nezlobinsky, A. Okenov, A. V. Panfilov

**Affiliations:** 1 Department of Physics and Astronomy, Ghent University, Krijgslaan 281, 9000 Gent, Belgium; 2 Ural Federal University, Ekaterinburg, Russia

**Keywords:** Cardiology, Computational models

## Abstract

One of the important questions in cardiac electrophysiology is to characterise the arrhythmogenic substrate; for example, from the texture of the cardiac fibrosis, which is considered one of the major arrhythmogenic conditions. In this paper, we perform an extensive *in silico* study of the relationships between various local geometric characteristics of fibrosis on the onset of cardiac arrhythmias. In order to define which texture characteristics have better predictive value, we induce arrhythmias by external stimulation, selecting 4363 textures in which arrhythmia can be induced and also selecting 4363 non-arrhythmogenic textures. For each texture, we determine such characteristics as cluster area, solidity, mean distance, local density and zig-zag propagation path, and compare them in arrhythmogenic and non-arrhythmogenic cases. Our study shows that geometrical characteristics, such as cluster area or solidity, turn out to be the most important for prediction of the arrhythmogenic textures. Overall, we were able to achieve an accuracy of 67% for the arrhythmogenic texture-classification problem. However, the accuracy of predictions depends on the size of the region chosen for the analysis. The optimal size for the local areas of the tissue was of the order of 0.28 of the wavelength of the arrhythmia. We discuss further developments and possible applications of this method for characterising the substrate of arrhythmias in fibrotic textures.

## Introduction

Cardiac arrhythmias remain a major public health problem which can lead to sudden cardiac death or heart failure, and are associated with substantial morbidity and mortality from other causes^1^. One of the best-known arrhythmogenic conditions is fibrosis of cardiac tissue, i.e., the presence of a large number of inexcitable cells: fibroblasts or myofibroblasts^2^. Such cells disturb normal wave propagation and lead to the formation of wavebreaks and cardiac arrhythmias. Fibrosis presents in most forms of heart disease, e.g., after myocardial infarction, during heart failure, during sustained atrial fibrillation, and under many other pathological conditions^3,4^. Fibrosis also increases with age, which contributes to a higher propensity to arrhythmias in older people^2^. Therefore, understanding mechanisms of cardiac arrhythmias due to fibrosis is a broad area of research ranging from direct clinical studies to experimental *in vitro* and *in vivo* research. Also, a lot of attention has recently been paid to modelling approaches. This is because modelling allows the researcher to test different hypotheses on the mechanisms with the possibility of complete control over the properties of fibrotic tissue.

In modelling of fibrosis there is also a wide variety of approaches, ranging from patient-specific models of the heart with fibrosis data obtained from LGE-MRI imaging^5^, to various studies of fibrosis at tissue level^6–9^, to detailed studies of wave propagation using cell-based models^10,11^. Those studies investigate various aspects of wave propagation in fibrotic tissue. However, the central questions here are to understand how, why and when fibrosis leads to arrhythmias. Here, one of the widely used modelling approaches is to generate various fibrotic textures and study how each texture affects the onset of arrhythmias.

An important study of this type was reported in a paper by Alonso et al.^12^. The authors showed that the highest probability of arrhythmias occurs when wave propagation is close to the percolation threshold, i.e., when wave propagation is almost blocked due to the presence of the fibrosis. Their second finding was that the probability of arrhythmias was strongly correlated with the probability of the formation of fibrotic clusters above a typical size. This is an important result, as it relates geometric features of fibrosis to arrhythmias. Another study^13^ showed that probability of arrhythmias depends on the heterogeneity of fibrotic texture. Other studies^6,9^, although they did not target the process of arrhythmia onset directly, show that, depending on texture, fibrosis can induce anisotropy in wave propagation and substantially increase the path of the wave due to zig-zag propagation^9,14^, which can increase the probability of arrhythmias. However, which of the above-listed features of fibrosis are the most important for the onset of arrhythmias, and whether it is possible to distinguish arrhythmogenic from non-arrhythmogenic textures based on these features, remain largely under investigation.

The aim of the current study is to perform large-scale simulations of the process of formation of arrhythmias in fibrotic texture, to find arrhythmogenic and non-arrhythmogenic textures, and to compare both of them by various geometric features of fibrosis, such as clusters, their positions, local density, zig-zag propagation, etc. Further, we test which of them, and which of their combinations, can be the best predictors of arrhythmogenicity. We performed, in total, about 9000 simulations using the Aliev-Panfilov model of cardiac tissue^15^, and analysed the listed features in 4363 arrhythmogenic and 4363 non-arrhythmogenic textures. We show that it is possible to separate arrhythmogenic and non-arrhythmogenic textures; however, it strongly depends on the size of the texture. From the studied features, those related to cluster sizes and their positions have the greatest significance; however, other features also improve the accuracy of the prediction. Overall, we obtained an accuracy of prediction of around 65–67%, what we consider to be adequate for the initial study. However, we think it can be improved in subsequent research by introducing additional features of the fibrotic textures.

## Methods

### Cardiac models

To form our training dataset, we used the Aliev-Panfilov cardiac model, a reaction-diffusion system of two equations in a 2D isotropic medium formulation:

1 $$\begin{aligned} \frac{\partial u}{\partial t} = D \nabla ^{2} u - ku(u-a)(u-1) - uv, \frac{\partial v}{\partial t} = -\left(\epsilon + \frac{\mu _{1}v}{u + \mu _2})(v + ku(u - a - 1)\right), \end{aligned}$$

where *u*—is the transmembrane voltage; $D=1$—is the diffusion coefficient for isotropic medium; $k = 8$; $a = 0.1$; $eps = 0.01$; $\mu _1 = 0.2$; and $\mu _2 = 0.3$.

Calculations were performed using the approach^16^. In particular, we applied explicit finite-difference method and to evaluate the Laplacian in Eq. (1) used 5-points stencil^16^. The spatial discretisation step was 0.1, and the time step was 0.0015.

In our model, a typical period of arrhythmia was 26 time units. Thus, if we assume that it corresponds to 260 ms (typical of arrhythmias in fibrotic tissue studies in ionic models^13^), we find that one time unit in our model corresponds to 10 ms. Subsequently in the text we will use ms to indicate model time. For spatial units, we note that the typical wavelength of the arrhythmia in our model ($\lambda$) is about 200 grid points. We scale by $\lambda$ the size of the tissue. For example, tissue of the size $256\times 256$ grid points will have the size of $1.28\lambda \times 1.28\lambda$.

Boundary conditions were the no flux through the boundaries:

2 $$\begin{aligned} {\vec{n}} \nabla u = 0, \end{aligned}$$

where ${\vec{n}}$—is the normal to the boundary.

Points assigned as fibrotic were treated as non-conductive obstacles with the no flux through the boundaries. To setup boundary conditions we applied the following procedure. The Laplacian in Eq. 1 account for divergence of currents to a given point from its neighbours. For the inner point, i.e. a point for which all neighbours are inside the medium it gives a standard 5-points stencil. However, if at least one of the neighbors was outside the medium, or was a fibrotic cell we put a current from this point equal to zero and thus correspondingly modified the weights for the calculation of the Laplacian.

### Stimulation protocol

The stimulated area was the upper side of the medium. At the time of stimulation the voltage at 7 upper rows of the medium was set to maximal value ($u=1$). Arrhythmia initiation requires a high-frequency passing of fibrosis textures. In simple 2D modelling, equidistant time intervals are usually used; however, a Wenckebach effect may lead to a spontaneous spiral waves initiation near the electrode which is not related to a texture features. Here, we used an adaptive stimulation protocol, similar to that used in clinical procedures^17^. In particular we constantly monitor variable *u* at 7 upper rows of the medium. We wait until the variable *u* decreases below $u < 0.01$, as it indicates when the tissue there will be recovered from the previous excitation. After that we wait 6 time units, which corresponds to 60 ms, and deliver a new stimulus. This allows us to prevent the onset of spirals at the electrode. We applied such stimulation for 230 time units. During that time stimuli were normally applied. After that we continued stimulation for additional 170 time units during which we observed stabilization or disappearance of the spiral wave.

### Fibrosis textures

In this study, we modelled a myocardial texture as a 2D binary $256\times 256$ matrix which contains only two types of cells: 0 (excitable myocardial element) and 1 (non-excitable fibrotic element). The fibrotic elements are distributed uniformly using the Mersenne Twister pseudo-random number generator. Each element of the matrix has probability *p* to become fibrotic, where *p*—is the given density of fibrosis in the matrix. Thus, for a fibrosis density of 33%, about 33% of the matrix elements will be fibrotic. The fibrotic elements formed a texture which contained individual fibrotic cells and fibrotic clusters which were analyzed in our paper.

### Core of spiral wave

**Figure 1:**
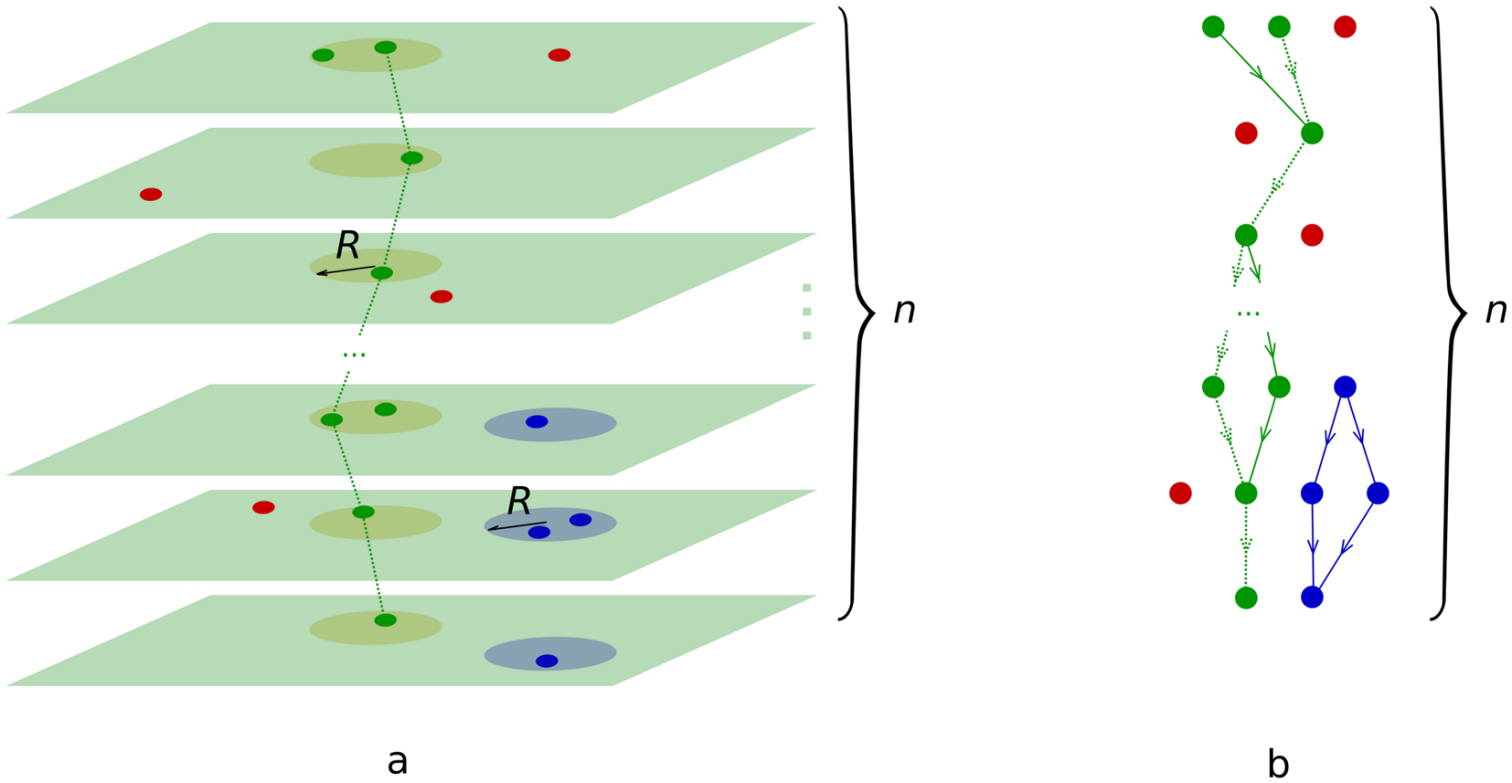
Graphical representation of the graph-based automatic spiral wave core recognition algorithm. Sets containing wavefronts’ intersection points are represented as: (**a**) collection of 2D maps; (**b**) directed graphs. Green is the major (or core) group that contains points from all *n* sets. Blue is the minor group that contains points from only a few neighboring sets. Red is isolated points that are not attributed to any group. *R* is the arbitrarily chosen radius for dividing points into groups. The green dotted line (shortest path from the first set points to the last set points) corresponds to the core trajectory.

The algorithms use the graph approach similar to^18^. To find the cores of the spiral waves, we built directed graphs containing all existing trajectories of the wavefront movement (Fig. 1). We assigned ($n+1$) transmembrane potential maps with spiral waves from 0 to *n* according to their timestamps. For all ($i \in {0,\ldots ,n-1}$) maps, we found coordinates (*x*, *y*) of points where the wavefront intersects with the wavefront from the neighboring ($i+1$) map. Points from collected *n* sets were grouped according to the Euclidean distance between them (Fig. 1a). The group was labeled as major (or core) if it contained points from all *n* sets, or else it was labeled as minor. Groups can be treated as directed graphs with vertices constructed from points, and directed edges that connect points from neighboring sets (Fig. 1b). The Euclidean distances between connected points are weight-assigned to the corresponding edges. By applying Dijkstra’s algorithm to the major group graph, we found the spiral wave core trajectory. Similarly, we can find unstable cores from minor groups by specifying the minimal number of maps ($m < n$) where unstable cores should be represented. Points that are not attributed to any core trajectories can be explained by fibrosis effects, such as wavefront distortions or raptures.

### Local textures marking

‘Local texture’ means the specific region of the original mesh to be analyzed. Every local texture in our study is denoted as a square of a variable size ($24\times 24$, $40\times 40$, $56\times 56$, etc.) and may be marked with two possible classes: arrhythmogenic (contains the spiral wave core) and non-arrhythmogenic (no spiral wave core). Knowing the location of the core of the spiral wave inside the texture of the original size ($256\times 256$), we can start the procedure of local textures extraction. We are considering two groups of local textures: arrhythmogenic and non-arrhythmogenic textures. The first group can be cut from/around the location of the spiral wave core, where the center of the texture corresponds to a center of the core’s trajectory. The local texture can be of any size. Non-arrhythmogenic local textures with the same size can be taken from textures where cores were not observed.

**Figure 2:**
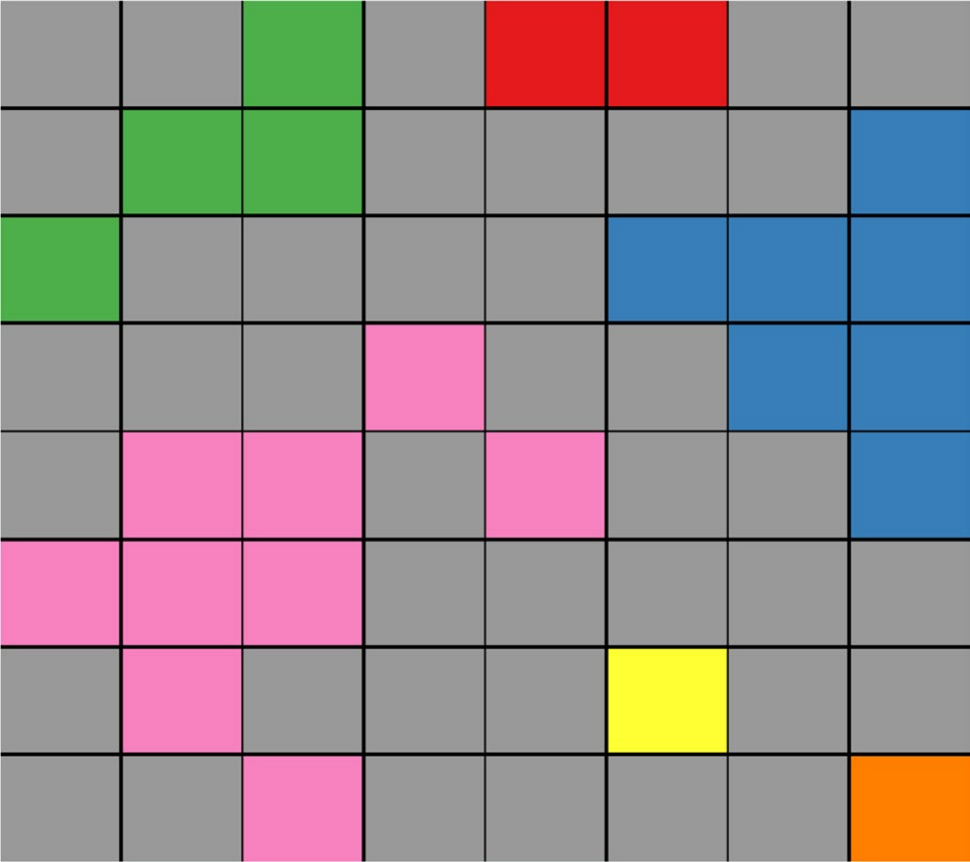
Clusters extraction example. Gray color indicates empty space; other colors show different clusters marked on the $8\times 8$ grid.

### Features extraction

#### Fibrotic clusters

Every local texture can be represented as a distribution of connected clusters. We labeled all connected fibrotic elements as one cluster: we start from the first matrix element marked as fibrotic and look for neighboring fibrotic elements which are directly connected with the target element (eight-points stencil). Those elements as well as the marked element will be assigned to the same cluster, etc (Fig. 2).

Similarly, we mark all clusters using the same connected elements search.

**Figure 3:**
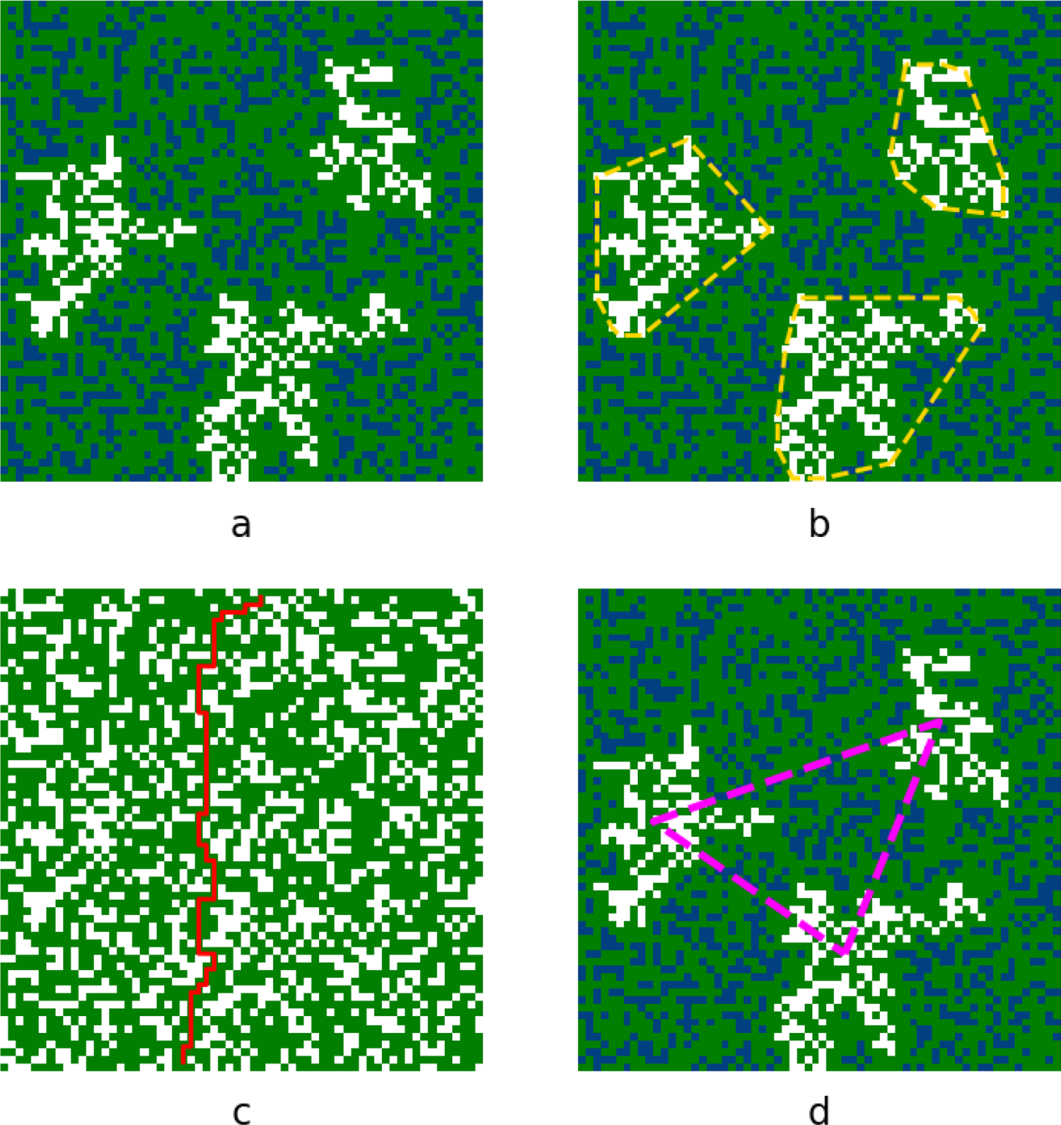
Studied features of fibrosis illustrated in a local texture ($64\times 64$ size): Three largest clusters (**a**); solidity (**b**); zig-zag propagation (**c**); centroids mean distance between three clusters (**d**).

#### Cluster parameters

We consider three geometrical parameters corresponding to cluster features:

1. Number of points in the cluster denoted as a cluster’s area.
2. Ratio between the cluster area and the convex hull area of the cluster, denoted as a solidity. ‘Convex hull’ is the smallest convex polygon that surrounds all elements of the cluster.
3. A spatial characteristic of a group of clusters, calculated as a distance between the clusters’ geometrical centroids.

#### Zig-zag propagation

We use the length of the shortest path between the opposite sides of the texture as a characteristic of a texture’s conductivity. Here we use a primitive cellular automaton to build an undirected graph for initially activated cells starting from the source point on the texture’s border, ending in the first activated cell on the opposite border. For each local texture, we compute 10 trajectories for different source points and find a mean path length as a zig-zag propagation feature.

### Metrics calculation

To estimate the k-NN model quality, we used the generally accepted analysis metrics: accuracy, precision, recall and f1-score^19^.

3 $$\begin{aligned}&Accuracy = \frac{TP + TN}{TP + TN + FP + FN}, Precision = \frac{TP}{TP + FP}, \nonumber \\&Recall = \frac{TP}{TP + FN}, F1 = \frac{2TP}{2TP + FP + FN} \end{aligned}$$

where TP—true positive (true arrhythmogenic) cases, TN—true negative (true non-arrhythmogenic) cases, FP—false positive cases and FN—false negative cases.

## Results

### Global versus local fibrosis textures

**Figure 4:**
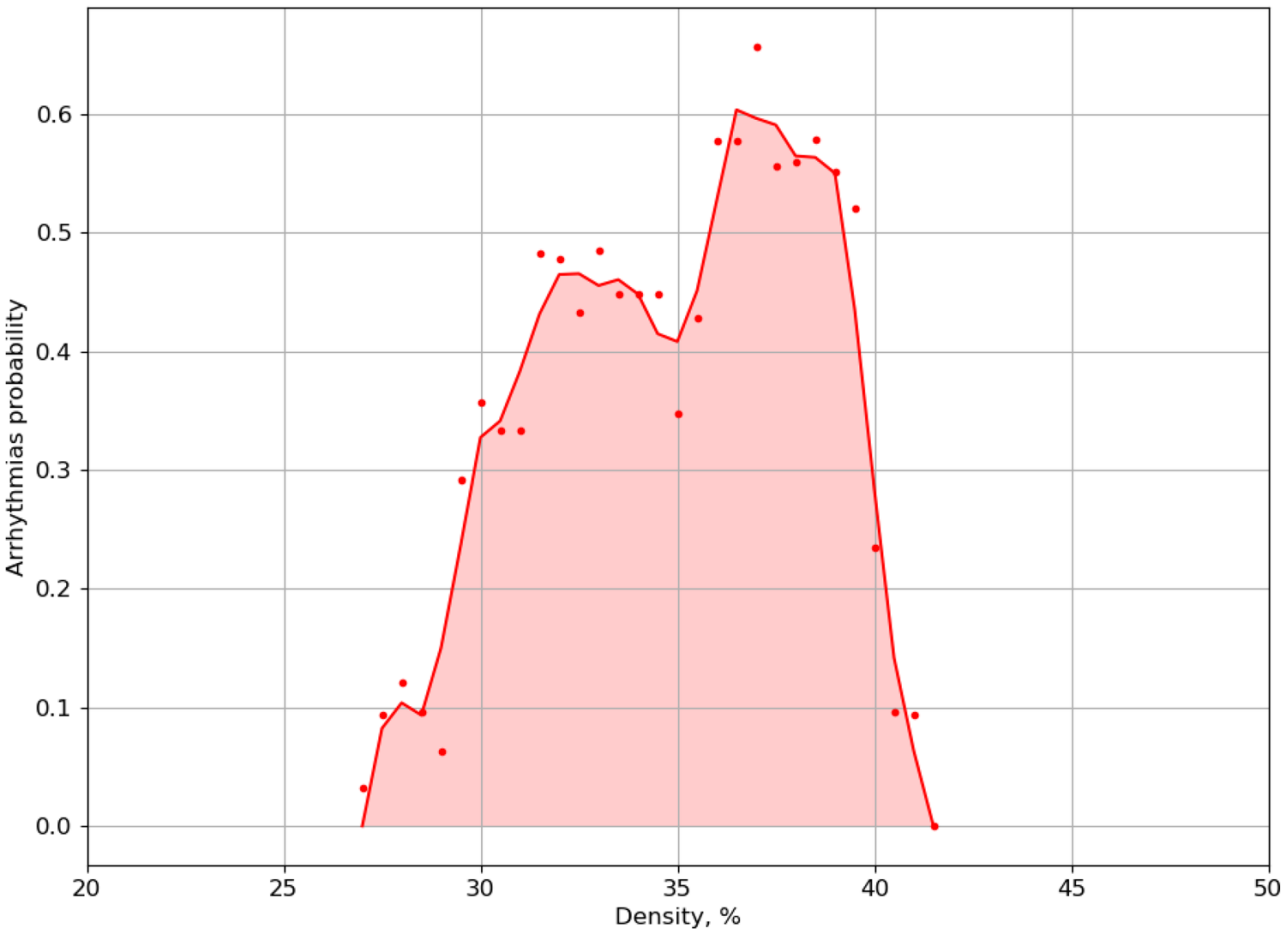
Arrhythmia initiation probability versus fibrosis density. Each value represents mean probability of arrhythmia for 30 calculations with unique fibrosis matrix of the same density. Calculations were performed for 30 density values (red points) in the range from 27 to 41.5%, thus in total for 900 textures.

We studied the process of arrhythmia generation by external high-frequency stimulation in fibrotic textures. It is well known that probability of arrhythmia strongly depends on the fibrosis percentage. Figure 4 shows such dependency in our model. We see that the probability is almost zero for fibrosis percentage < 28%. It reaches maximum at 37%, and then decreases. Our aim is to find which features of the fibrotic texture are most important for the onset of arrhythmia, and thus separate arrhythmogenic from non-arrhythmogenic textures. To better account for such separation, we decided to study textures with 33% fibrosis, since in that case we have a 48% probability to generate arrhythmias with the stimulation protocol used in our study. Therefore, we obtained almost equal sets of possible arrhythmogenic and non-arrhythmogenic textures.

**Figure 5:**
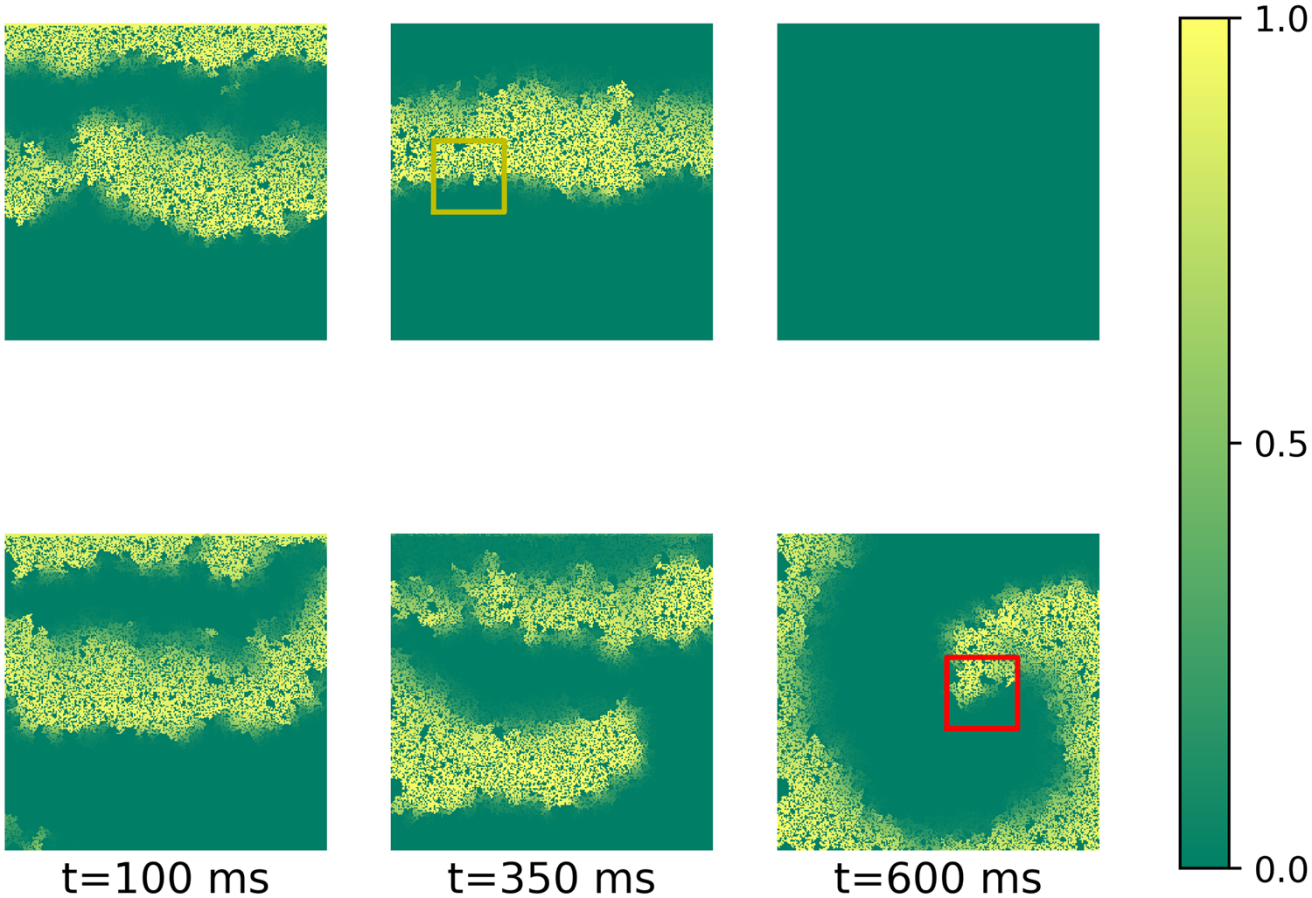
Initiation of arrhythmia by high-frequency pacing in the non-arrhythmogenic texture (the upper row) and in arrhythmogenic texture (the lower row). The local arrhythmogenic (red) and non-arrhythmogenic (yellow) textures ($56\times 56$) are marked on the frames.

For our study, we generated about 9000 unique 2D textures of 33% fibrosis and tried to initiate arrhythmia using our stimulation protocol.

Figure 5 shows an example of a typical simulation. It shows propagation of waves generated by two successive stimuli. The upper row shows non-arrhythmogenic texture, when both waves eventually disappear at the boundary. The lower row shows arrhythmogenic texture, when external stimulation initiates sustained arrhythmia. Arrhythmia was considered as sustained if we obtained at least one spiral wave which remained stable until the end of calculation (4000 ms or approximately 5 cycles after the end of stimulation).

We obtained no arrhythmia in 4419 textures and arrhythmia in 4363. Our first question was to study if there is any difference between arrhythmogenic and non-arrhythmogenic textures in terms of features identified in the previous studies based on fibrotic clusters characteristics^12^, heterogeneity in the density of the fibrosis^13^, and zig-zag propagation^14^. In particular, we analysed a set of features belonging to five groups of characteristics: cluster area (Fig. 3a) for the one, three or five largest clusters in a texture; cluster solidity (ratio of cluster area to cluster convex hull area) for the same number (one, three or five) of extracted clusters (Fig. 3b); zig-zag path length (Fig. 3c); and the distance between three largest clusters in a texture (Fig. 3d).

**Figure 6:**
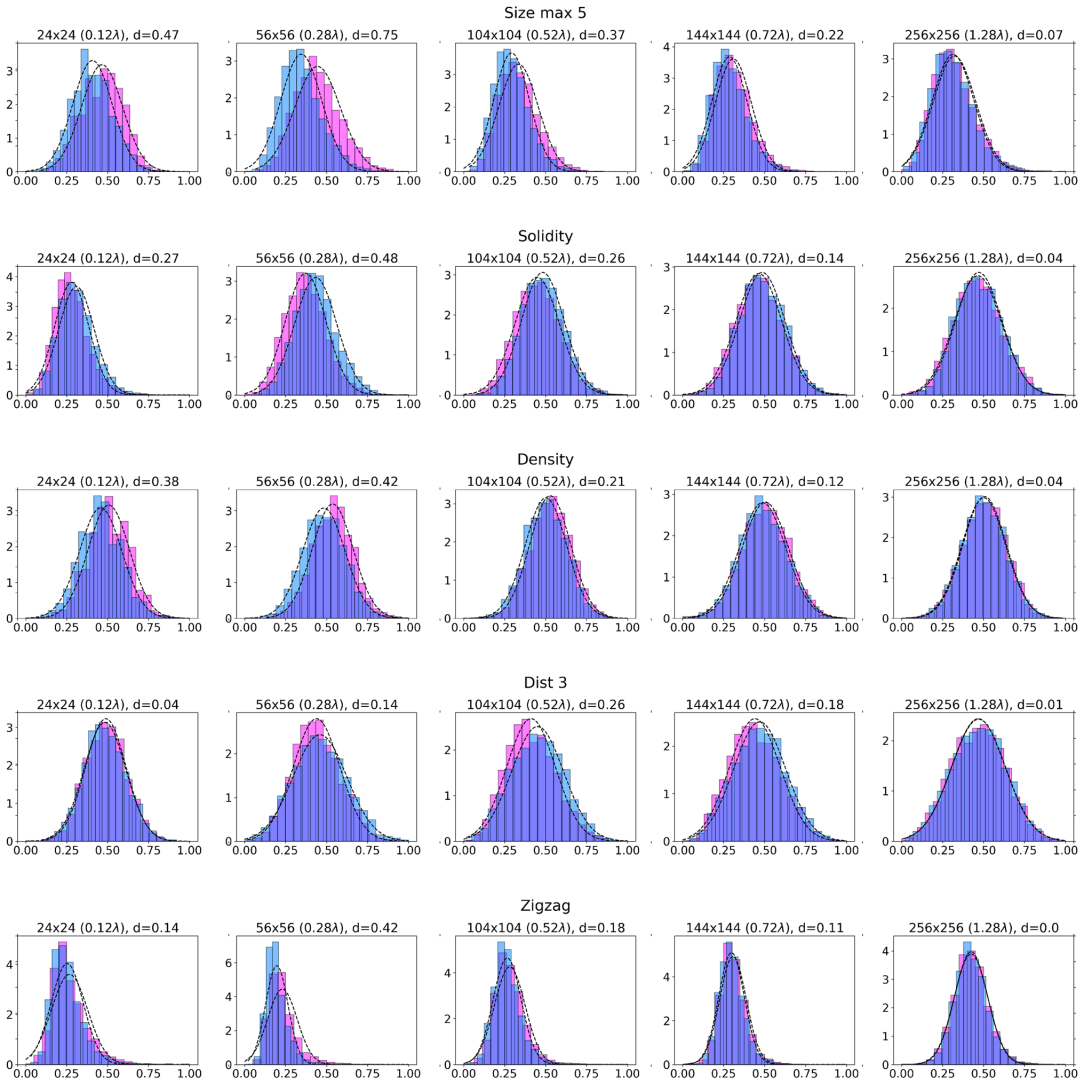
Distributions in arrhythmogenic and non-arrhythmogenic groups for five features for different textures’ sizes. Cohen’s *d* value estimates the difference between the two groups of distributions. All basic features used in the study are presented: cluster area (size), specifically for five largest clusters; solidity for one largest cluster; fibrosis density; centroids’ distance (three clusters); and zig-zag propagation path length. For this and the following figures, we use the entire dataset of 8726 textures.

We started with the original-size textures ($256\times 256$ or $1.28\lambda \times 1.28\lambda$), which we call the global textures. As the density of fibrosis was a constant 33%, we did not use the feature ‘density’ for the analysis of global textures. We found that distributions of all features for arrhythmogenic and non-arrhythmogenic groups can be well approximated by Gaussian curves. However, for global textures, all distributions for arrhythmogenic and non-arrhythmogenic groups are visually non-distinguishable from each other (Fig. 6, right column). We also see it from the Cohen’s *d* values, which are all below 0.1.

As the next step, we analysed not whole $256\times 256$ textures, but regions of the texture where the arrhythmia source was located. Fig. 5 ($\hbox {t}=600$ ms) shows an example of such regional texture with the size $56\times 56$ ($0.28\lambda \times 0.28\lambda$). Here, the arrhythmogenic region (a square region where the core was located) is marked by the red square. Note that we did not do any additional simulations to induce arrhythmia in such small regions, and just found them by identifying the location of arrhythmias in the same $256\times 256$ textures. In total, we extracted 4363 local arrhythmogenic textures from 4363 global ($256\times 256$) textures marked as arrhythmogenic. If the global textures contained several arrhythmia sources, we selected the first one detected by our graph-based algorithm. This guaranteed us the independence of all local textures from each other. We also chose 4363 non-arrhythmogenic local textures, randomly, from non-arrhythmogenic $256\times 256$ textures.

We varied local texture sizes from $16\times 16$ ($0.08\lambda \times 0.08\lambda$) to $256\times 256$ ($1.28\lambda \times 1.28\lambda$) with a step of $8\times 8$, gradually increasing the texture size and estimating the distribution’s effect size. We see (Fig. 6) that, in contrast with global textures, when the size of the texture decreases we start to observe a clear separation of arrhythmogenic and non-arrhythmogenic textures for all used characteristics. However, a further decrease in size makes the separation worse. To estimate the extent of that difference, we used Cohen’s *d* value and its standard interpretation^20^. We suggest that distributions for our group have enough normality to apply Cohen’s *d*, and its variances are close enough.

Figure 6 shows that for all the features, Cohen’s *d* initially increases, reaches some maximal value, and then decreases again. For cluster area, solidity, density and zigzag, Cohen’s *d* optimal value reaches 0.4–0.7 around the local textures size of $56\times 56$ ($0.28\lambda \times 0.28\lambda$) compared to ${d}=0.00{-}0.07$ for the global textures $256\times 256$ ($1.28\lambda \times 1.28\lambda$)^20^. A similar effect may be observed for centroids’ distance (dist 3), but *d* value reaches its optimum around $104\times 104$ ($0.52\lambda \times 0.52\lambda$). For most of the considered features, if we are far above or below the size of the core of the spiral wave, *d* value increases and it becomes harder to distinguish arrhythmogenic and non-arrhythmogenic textures.

Thus, for our further study, we chose the local texture size of $56\times 56$ and continued our analysis.

### Local fibrosis textures analysis

Our next step was to study what is the predictive power of each of the selected characteristics and all of them combined. For that, we applied the *k*-nearest neighbors (*k*-NN) algorithm, which is a simple and widely used way to build a predictive model. The *k*-NN model was trained and validated using five-fold cross-validation for the optimal *k* parameter, which represents the number of neighbors that are taken into account. The *k* parameter for all the features and their combinations was always within the range 100–200.

We started with one-feature analysis. Here, the *k*-NN algorithm automatically defines the optimal threshold for separation of arrhythmic and non-arrhythmic textures.

**Figure 7:**
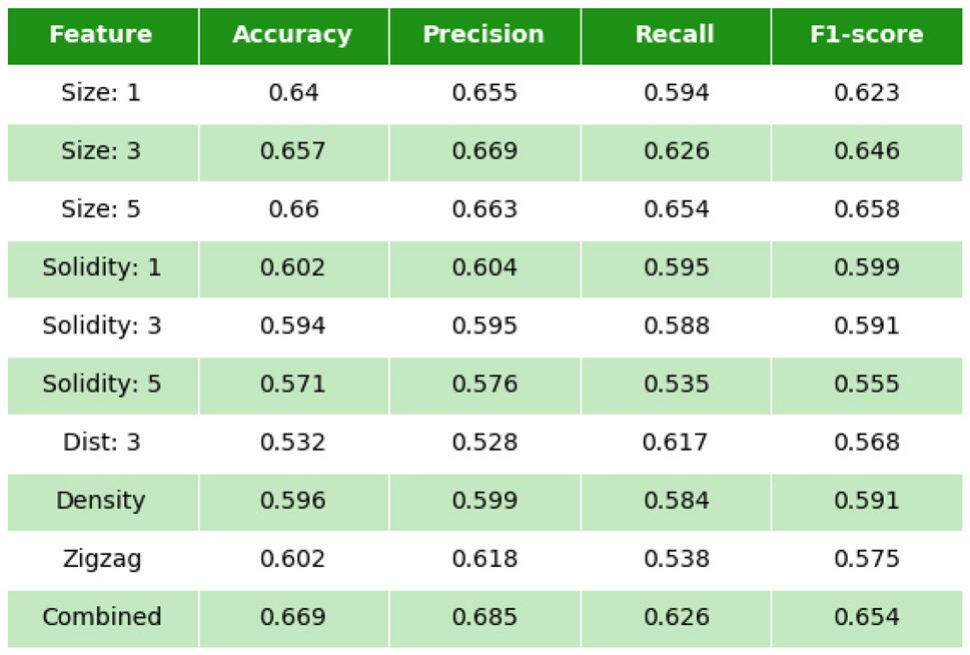
Accuracy, precision, recall and f1-score for features used in the study. All values correspond to $56\times 56$ local textures.

Using *k*-NN, we found the following standard characteristics of the predictive power of each corresponding feature (Fig. 7). The accuracy (fraction of arrhythmogenic and non-arrhythmogenic local textures which are predicted correctly [true arrhythmogenic and true non-arrhythmogenic respectively]), precision (fraction of true arrhythmogenic local textures among all local textures predicted as arrhythmogenic), and recall (fraction of true arrhythmogenic local textures which were predicted correctly among all true arrhythmogenic local textures in the dataset). (More details on statistical definitions can be found^19^).

**Figure 8:**
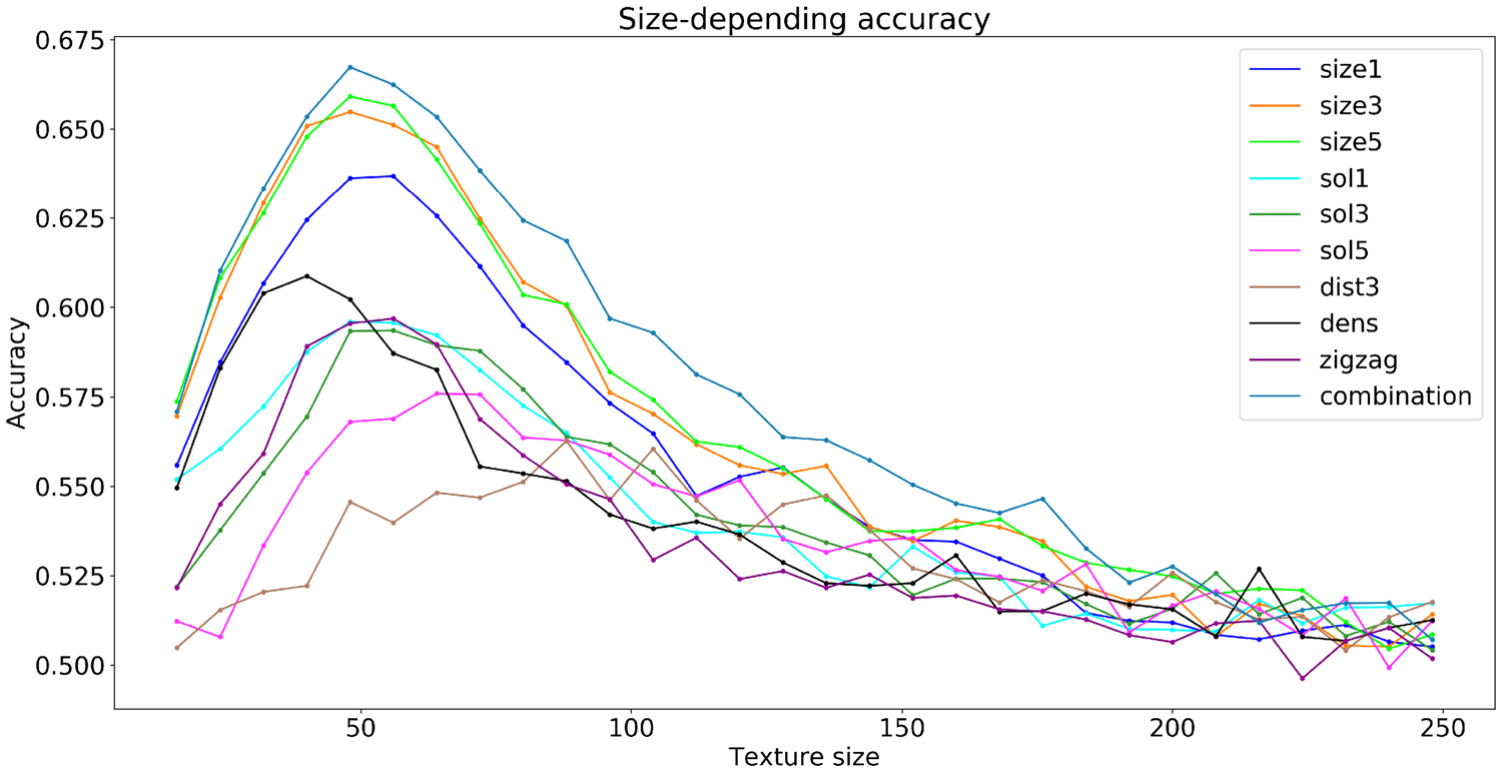
Features accuracy depending on the textures size. Combination corresponds to all the features listed in the legend above, united in one feature vector.

According to our analysis, cluster area is the most prominent feature, which gives the main contribution to the prediction outcome (around 65%). We see that the prediction based on the largest cluster gives almost the same quality as that based on the mean area of three or five largest clusters (66%).

It is important to note that in a local texture, maximal cluster size is the relative value represented by the largest structure in the local texture itself ($56\times 56$), but not in the original texture ($256\times 256$).

Solidity is another interesting feature, representing the ratio between the area of the cluster and the area of the convex hull of the cluster. A cluster’s solidity has a relatively small Pearson correlation coefficient (0.3–0.4) with the cluster’s size, but also has a good predictive ability in one-feature analysis (57–60%). Here, we also checked one, three or five mean solidity values for the one, three or five largest clusters, respectively, to find if there is an effect with neighboring clusters.

**Figure 9:**
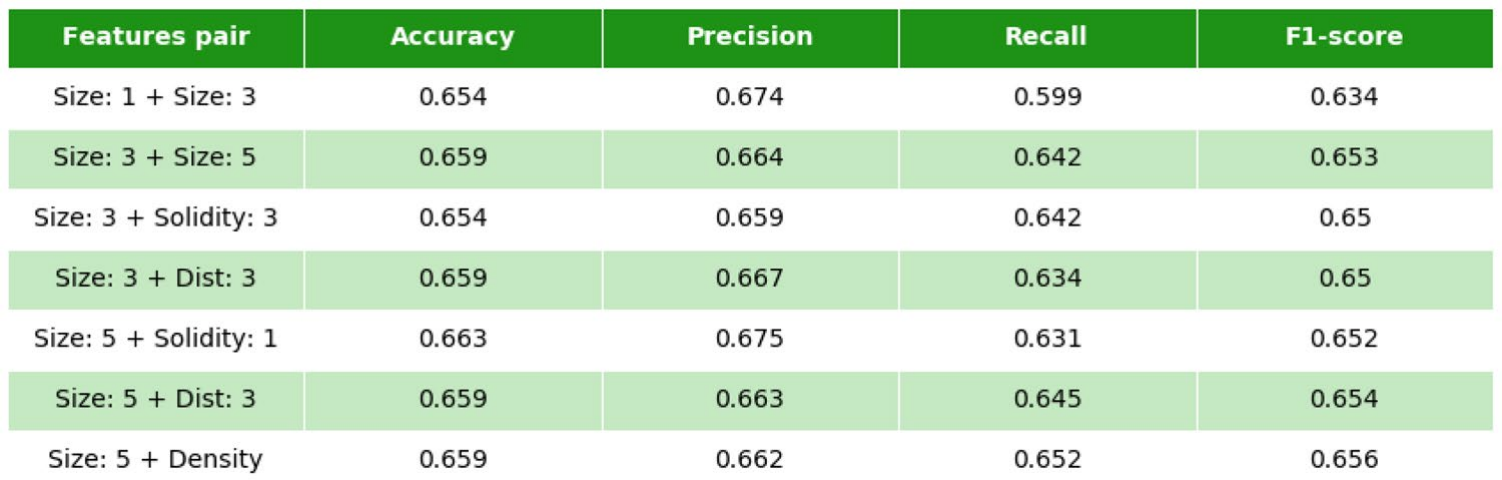
Accuracy, precision, recall and f1-score for pairs of features. All values correspond to $56\times 56$ local textures.

Effective conductivity properties of local textures estimated with zig-zag propagation show accuracy of about 60%, and a Pearson correlation with the largest cluster size is about 0.45. Spatial distribution of the largest clusters can be described as the mean distance between their geometric centres (centroids). This feature has no correlation with the largest size (Pearson correlation <0.1), which seems to be good additional information for the analysis. The last feature used in our study is density’a proportion between the fibrotic and healthy cells. Though the density of the whole structure is 33%, the density in local textures has fluctuations. The predictive value of density was about 59%. Finally, we used all features in combination and found that it results in 2% better accuracy of prediction of arrhythmogenicity of the texture than any of the single characteristics alone. Here, of course, the most important contribution comes from the characteristics based on the cluster area. Thus, if in addition to cluster area, we use one or two more features (solidity, distance, zigzag or density), we also obtain accuracy close to the 67% (66–67%) achieved by using all parameters combined (Fig. 9).

We also studied how the prediction accuracy behaviour depends on a texture’s size (Fig. 8). We see that for different characteristics, the accuracy reaches its maximum for a different size of the texture. However, in most of the cases, it peaks for sizes between $40\times 40$ and $60\times 60$. Also, the location of the maximum corresponds to the size where a Cohen’s coefficient *d* has maximum value (Fig. 6). The accuracy decreases when there is a high overlap of the group’s distribution.

**Figure 10:**
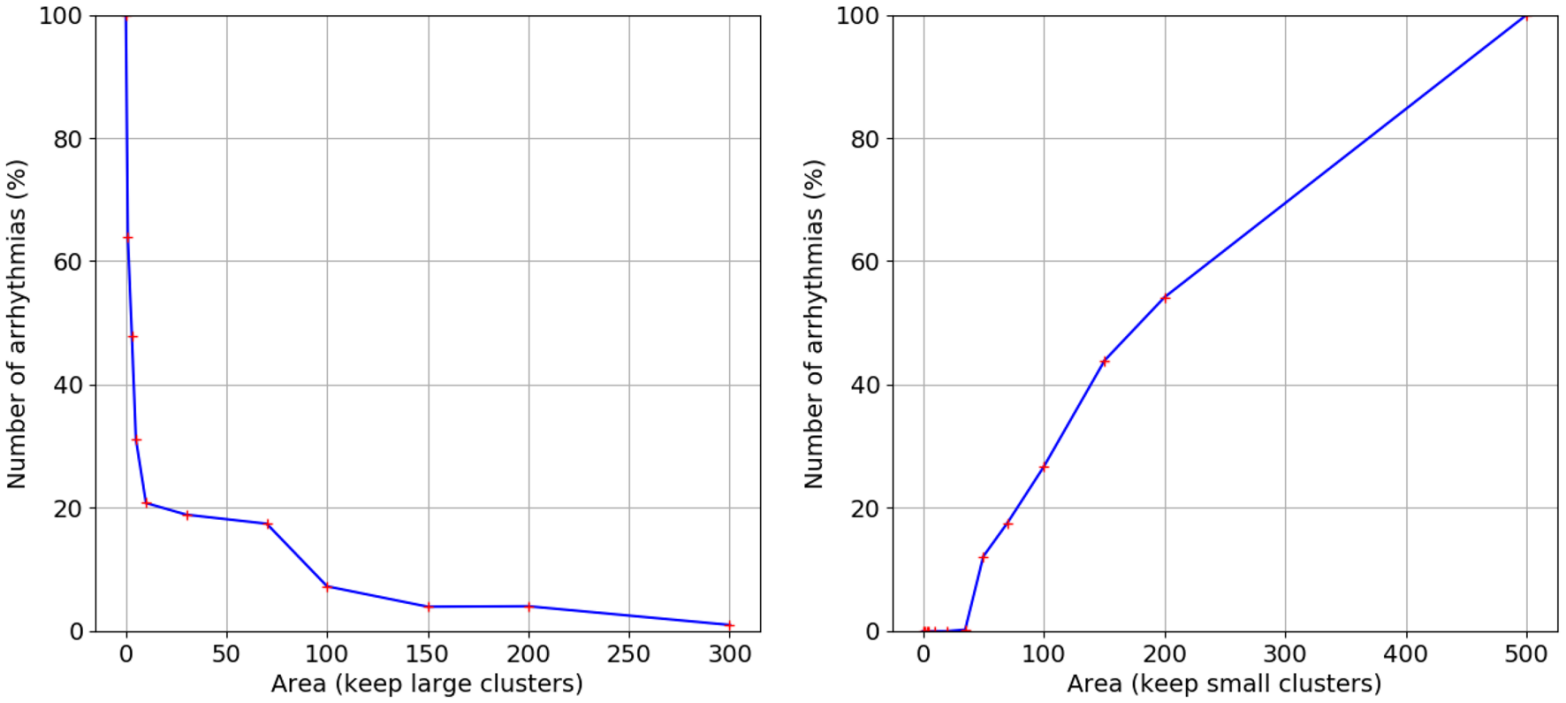
Number of arrhythmias for 500 filtered textures. All textures were initially arrhythmogenic, but lost this feature as the texture was filtered. The x-axis shows cluster area more (**a**) or less (**b**) than the threshold.

Given our finding that largest cluster is the most important parameter, we also studied if the clusters alone are sufficient for the arrhythmia onset. For that, we performed additional simulations in which, in global arrhythmogenic textures, we filtered fibrosis based on cluster sizes. We considered two ways of filtering: from below, i.e., when we remove from the texture all clusters which are below the given size (Fig. 10a), or from above, i.e., when we remove clusters whose size is larger than the given threshold value (Fig. 10b). We see that in spite of the importance of cluster area, both large and small fibrotic textures are important for arrhythmia onset. Indeed, if we remove fibrotic clusters with the area of just two cells and one cell, the probability of onset of arrhythmia decreases more than 50%. Interestingly, with further removal, the graph almost instantly reaches a state at the level of 20%, and then gradually decreases with the area. It indicates that we have at least one arrhythmia in five which is not directly related to the diffuse fibrosis, where the arrhythmia is probably generated due to global cluster geometry. However, we also see that removal of large clusters dramatically decreases the probability of the arrhythmia, and it drops to zero in the absence of large clusters. Thus, we see that global clusters are important for the arrhythmia onset, but small-scale fibrosis is also extremely important.

**Figure 11:**
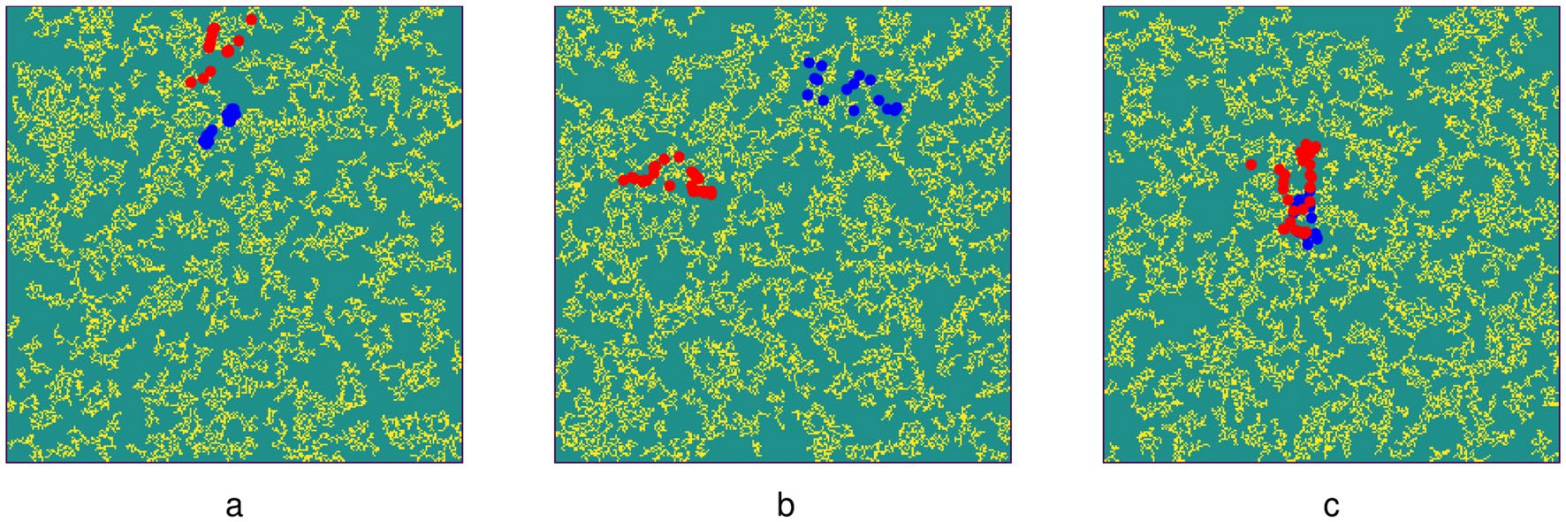
Examples of location of arrhythmias before and after filtering the texture for small clusters. The blue points show location of arrhythmia in non-filtered texture, the red point show the location of the arrhythmia in texture filtered for clusters with the size < 20. The filtered texture is represented by the yellow color. Case (**c**) shows almost the same position of the spiral wave rotation before and after filtration, while cases (**a**) and (**b**) show different positions of the arrhythmias in the texture.

Arrhythmias remaining after filtration differ from the arrhythmias which occur in non-filtered textures. We compared the sites of initiation of arrhythmias in 86 textures, in which it was possible to induce arrhythmias after clusters with the size < 20 were removed. Fig. 11 shows typical examples of location of arrhythmias before (blue) and after filtering (red). In most of the cases (70–80%) the location of arrhythmia changes, as can be seen in Fig. 11a, b. And only in about 20–30% the arrhythmias in filtered and unfiltered textures occur at the same location, as in Fig. 11c. This indicates that in most of the cases arrhythmias were not formed at large clusters, however, these clusters are important for maintenance of arrhythmias, as was shown above. Also, different place of formation of arrhythmia in absence of small scale fibrotic clusters indicates that in the same texture we probably can have multiple places where the arrhythmia can be formed, which may or may not reveal themselves depending on the environment, such as diffuse fibrosis.

Our data presented in Fig. 10b indicate the existence of a critical cluster size, which is essential for existence of sustained arrhythmias. Figure (Fig. 10b) shows that arrhythmias are completely absent, if clusters with area > 50 points are filtered out. The probability of arrhythmias reduces by 50% if clusters with area > 180 points are removed. This observation is consistent with Alonso et al.^12^, where the correlation between the cluster size and the arrhythmia probability was also shown. Note, that in case of a square box an area of 180 grid points corresponds to a perimeter of approximately $0.3\lambda$. However, as clusters normally have a complex shape with jagged borders the perimeter can be substantially longer. It would be important to check whether the critical value of the cluster area depends on the cardiac model parameters and stimulation protocol or is more determined by the medium size and fibrosis texture.

## Discussion

In this paper, we performed large-scale studies of various geometric features of fibrotic textures and investigated if, by using them, we can separate arrhythmogenic and non-arrhythmogenic textures. For geometric features, we chose three main characteristics which were previously identified as important for the onset of arrhythmias: area of fibrotic clusters, local density of fibrosis and zig-zag propagation. We also introduced two additional characteristics based on fibrotic clusters: distance between cluster centres and ratio of the original cluster area to its convex hull area (solidity). We found that if the overall size of the texture was more than $120\times 120$ (or more than $0.6\lambda \times 0.6\lambda$—the wavelength of the arrhythmia), then none of the listed features was able to properly distinguish arrhythmogenic from non-arrhythmogenic textures (accuracy is about 50–55%). However, if we performed the same comparison for the textures with the size between $24\times 24$ and $80\times 80$ (i.e., between $0.12\lambda$ and $0.4\lambda$), then four out of five of the listed features were able to distinguish arrhythmogenic from non-arrhythmogenic textures with accuracy better than 55% (57–67%). The lower limit ($\lambda < 0.12$) is not surprising, since if the size of the texture is less than the core of the spiral, it should not affect its dynamics. However, the upper limit is less obvious. It probably indicates that in textures of a larger spatial size there are many non-arrhythmogenic sub-textures and few arrhythmogenic sub-textures which are essential for the spiral-wave onset. Thus the overall distribution is mainly determined by non-arrhythmogenic textures. If the size is large, then non-arrhythmogenic textures will determine the distributions for all textures, making arrhythmogenic textures non-distinguishable from the arrhythmogenic ones. In our dataset, the optimal size of textures to perceive differences was near $56\times 56$ ($0.28\lambda$), and it was used in our analysis.

Of studied characteristics, the most informative ones were based on the cluster area as suggested in^12^. If we use as the main feature just the area of the largest cluster, we obtain 64% prediction accuracy, which slightly increases if we use the three or five largest cluster areas. All other characteristics are less informative. However, if we make predictions based on all of them, the accuracy of prediction increases to 66.9%. It shows that such characteristics indeed provide independent, important information on the arrhythmogenicity of the textures. We found that almost the same quality of prediction can be achieved if we use just three characteristics (size max 5, solidity 1 and dist 3).

Note that overall accuracy of prediction turned out to be not very high. This can indicate that we probably do not yet know the most important arrhythmogenic characteristics of the tissue that can be more predictive. Probably, such characteristics should take into account not only the largest clusters, but also some features of diffuse fibrosis. This is because our simulations showed that if we remove diffuse fibrosis and small size clusters, the arrhythmogenicity of the textures is almost five times smaller. Also, the textures wrongly marked as non-arrhythmogenic may turn out to be arrhythmogenic if another stimulation protocol were used. This possible additional arrhythmogenicity would further increase the precision of the prediction of arrhythmogenic textures. We view our research as only the first step in large-scale studies of possible arrhythmogenic features of the textures for which we formulated our approach and generated a large dataset of textures. Using this approach, any additional characteristics of textures can be incorporated and investigated, and its predictive power can be determined.

We show that both clusters of small size and clusters of large size are important for initiation and existence of arrhythmias, and if small or large clusters are filtered out the probability of arrhythmias is strongly decreased. Note, that filtering out clusters also decreases the percentage of fibrosis and thus the observed affect can be partially explained by that. However, the real situation is more complex. For example, if we filter out clusters for size < 20 in our data, then the percentage of fibrosis will on average drop from 33 to 19%. From Fig. 4 we see that for such percentage of fibrosis the probability of arrhythmia is almost zero. However, in our case it is 8–10%. Thus filtered structures have some additional properties except fibrosis percentage which are essential for arrhythmia formation. It would be interesting to identify it in a subsequent study.

However, non-excitable clusters may be in the form of compact fibrosis, which occurs as a result of scar formation, e.g. due to myocardial infarction. Recently a detailed study of onset of arrhythmias in the presence of infarction scar was performed in^21^. In that paper the authors used state of art computational tools and studied how infarct scar dimension, repolarization properties and anisotropic fiber structure of scar tissue border zone affects the onset of arrhythmias. As one of the conclusions, the authors suggest that a certain ‘critical’ left ventricular scar volume is necessary to induce arrhythmia. This observation is in line with our finding that geometric characteristics are important determinants for reentry generation in fibrotic tissue. Note, that study^21^ includes much more detailed representation of cardiac tissue, which includes bidomain equations, 3D tissue anisotropy and heterogeneity around the scar. Thus we can also expect that our results in some form can be extended to more detailed models of cardiac tissue.

Our study has several limitations and can be extended in many ways. Here, we did not change the average percentage of fibrosis which was always 33%, accounting for arrhythmogenicity of about 50%. This was because, in this paper, we wanted to develop a general strategy of comparison of arrhythmogenic and non-arrhythmogenic textures and did it using a large amount of data. Extension of such an approach to other percentages of fibrosis is straightforward and certainly very interesting. Indeed, if one were to study textures with lower arrhythmogenicity, it might actually improve the prediction power of the algorithms. There would be substantially fewer local textures responsible for the onset of arrhythmias, and they may have more significant differences from the non-arrhythmogenic textures. However, in that case, more simulations will be needed to generate the corresponding dataset.

Another possible extension is to apply such methods not only for diffusive fibrosis, but also for other fibrotic textures. In papers^9,22,23^, it was shown that interstitial fibrosis has a more significant effect on wave propagation velocity, anisotropy and zig-zag propagation. That may indicate that interstitial fibrosis is more arrhythmogenic, and it would be interesting to find out how the characteristics used in this paper would work in that case as well.

It will be interesting to perform studies using an ionic model for cardiac tissue, e.g., models for human cardiac tissue^24–26^. Although we expect that the main conclusion of our study will still hold, some quantitative changes are possible. This is because, in the low-dimensional models used in our study, the ratio of wavefront duration to the duration of the pulse is substantially higher than in ionic models, and it may affect the process of wave interaction with the fibrosis^6,9^.

In our simulations we used a mono-domain description as a model for cardiac tissue. Another accepted descriptions are the bidomain^27^ and a tri-domain representation which was specially developed to account for heterocellular myocyte fibroblast coupling^28^. It is definitely interesting to study the process of spiral wave initiation in fibrotic textures which are represented by these different models. In general, we expect that qualitatively our main results will hold. Indeed, we claim that the most important determinants for prediction of the arrhythmogenic textures are the geometrical characteristics. These geometrical characteristics, especially size of the cluster, is, in our view, a reflection of the minimal wavelength in cardiac tissue, or minimal size of the obstacle around which wave can rotate in a closed ring of cardiac tissue^29^. Such minimal wavelength, in turn, is a reflection of the refractory period in cardiac tissue. In bidomain and tridomain models the refractory properties of cardiac tissue are, of course, present. Therefore, we expect the geometric characteristics will still be the most important. However, the critical wavelength depends on the velocity of the wave, and the velocity can be influenced by the bidomain representation, or by the myocyte-fibroblasts coupling considered in a tridomain model. Thus the quantitative results in those models may differ from that in our study. Note, however, that in^30^ it was shown that effects of fibroblast-myocyte coupling differ depending on membrane potential, fibroblast membrane capacitance and fibrosis density. Thus fibroblast-myocyte coupling can further increase spatial heterogeneity and promote formation of the arrhythmias.

In our paper we use only geometric features of the fibrosis. Another way to characterize fibrosis, is using electrical recordings. For example intracardiac electrogram amplitude decreases with increased fibrosis density. In^31^ a hybrid approach was developed that combines *in silico* and clinical electrograms to train a decision tree classifier to characterize the fibrotic substrate. It would be interesting to use similar approach and test if some parameters of intracardiac electrograms can be used to predict the arrhythmogenic sites of fibrotic textures, and if there is a difference in intracardiac electrograms in arrhythmogenic and non-arrhythmogenic textures.

Another important extension of our study will be to apply our approach to 3D anisotropic cardiac tissue. Note, however, that this is a very challenging study due to several problems. First of all, the structure of fibrosis in 3D is largely unknown and possible excitation patterns which can occur there are not properly classified. One of very few studies in that area showed that even in the absence of rotational anisotropy of cardiac tissue there are non-trivial effect of the probability of arrhythmia generation on the thickness, even for thin tissue slabs^32^. Also it is not a priori clear how the geometric characteristics of fibrosis used in our paper should be extended to 3D. For example, if we consider size of a cluster, we can of course use a volume in 3D. However, rotation in 3D is a combination of wave rotation in 2D sections. Thus the area of a cluster in a 2D section seems to be also very important. However, in spite of all that difficulties, study of fibrosis in 3D remains a very important problem which should and will be addressed in the future research.

Our approach uses only geometric characteristics of texture, and does not directly take into account functional properties of cardiac tissue. The probability of onset of arrhythmias depends on properties of cardiac tissue. In our approach we have only indirect influence of that. Our results are presented in dimensional units which are scaled based on the wavelength of the reentry. Changes in properties of cardiac tissue, e.g. excitability, will change the wavelength, and thus will affect the result. However, it may be interesting to look for characteristics which more directly affect wave propagation and formation of arrhythmias in the fibrotic tissue.

In conclusion, we performed a large-scale study with the aim of finding geometric characteristics of fibrotic texture responsible for the onset of cardiac arrhythmias. We found that, from the studied characteristics, the most predictive characteristics are those associated with the area of the fibrotic clusters. However, the differences can best be seen for textures of the size around $0.28\lambda$. Additional characteristics that improve the prediction accuracy of the arrhythmogenic textures are the largest cluster solidity, density and zig-zag path length. Overall, we were able to achieve prediction with accuracy of 66.9%, which, so far, is not high, and thus identification of additional geometric features of fibrosis and functional characteristics of wave propagation in fibrotic tissue is necessary and can be performed on a dataset that was generated in our study.
